# ZSL Orchestrates Synaptonemal Complex Assembly as a Central Region Scaffold to Ensure Synapsis Fidelity and Crossover Control in Polyploid Meiosis

**DOI:** 10.1002/advs.202521496

**Published:** 2026-03-04

**Authors:** Miaowei Geng, Shaochen Jia, Fei Cao, Cuiping Liu, Heshuang Zhang, Gang Xu, Jixin Zhuang, Yashi Zhang, Can Liu, Yuhang Zhao, Zilin Guo, Xinjie Yuan, Jiaqing Yang, Lei Chu, Bowei Cai, Hu Zhao, Chao Yang

**Affiliations:** ^1^ National Key Laboratory of Crop Genetic Improvement Hubei Hongshan Laboratory Huazhong Agricultural University Wuhan China

**Keywords:** Genome stability, Meiosis, Recombination, Synaptonemal complex

## Abstract

The synaptonemal complex (SC) is essential for accurate homologous chromosome pairing, recombination, and segregation during meiosis. Although several core SC components have been identified in plants, the molecular mechanism coordinating their assembly remains poorly understood. Here, through a refined temporal transcriptomic analysis of another development in the allotetraploid *Brassica napus*, this study identifies ZYP1–SCEP1/2 Linker (ZSL) as a central element scaffold that bridges the transverse filament protein ZYP1 with the heterodimeric central element proteins SCEP1/2. It is shown that ZYP1 loading onto chromosomes occurs independently of ZSL and SCEP1/2, whereas ZYP1 is required for their recruitment. Loss of ZSL impedes SCEP1/2 recruitment but not vice versa, and *zsl* mutants completely lack continuous SC central region assembly, leading to synapsis failure and chromosome mis‐segregation. This study further demonstrates that ZSL directly interacts with both ZYP1 and SCEP1/2. These findings define a hierarchical assembly cascade of ZYP1 → ZSL → SCEP1/2 during SC formation. Furthermore, analysis of HEI10 foci and genome‐wide crossover (CO) mapping in *zsl* mutants reveals an ≈100% increase in both male and female COs, accompanied by a loss of interference and elimination of sex‐specific CO differences. Together, the results establish ZSL as a key molecular adaptor coordinating SC central region assembly and CO patterning, providing new mechanistic insight into meiotic fidelity and genome stability in polyploid species.

## Graphical abstract

A central molecular adaptor, ZSL, is identified that links transverse filaments ZYP1 to central element SCEP1/2 to drive synaptonemal complex assembly in *Brassica napus*. Loss of ZSL abolishes synaptonemal complex formation, disrupts meiotic chromosome segregation, and markedly increases crossovers, providing mechanistic insight into meiotic fidelity and genome stability in polyploid species.

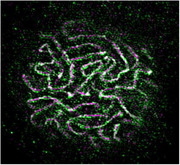

## 1 Introduction

In most of sexual reproductive organisms, meiosis relies on the synaptonemal complex (SC) to ensure faithful homologous interaction and accurate chromosome segregation, thereby maintaining genome stability and creating genetic diversity [1, 2, 3, 4]. The SC mediates synapsis to stabilize homologous pairing and regulates crossover (CO) formation. In polyploid species including the allotetraploid *Brassica napus* (*B. napus*, AACC, 2*n* = 38), which contain multiple copies of similar chromosome sets, the SC plays an additional critical role in facilitating diploid‐like meiotic behaviors: enforcing the pairing and recombination between true homologs while suppressing interactions between closely related homoeologs, preventing multivalent associations [5, 6, 7, 8]. Failure in this process leads to multivalent formation, unbalanced gametes, and reduced fertility [7, 8].

The SC is a tripartite protein structure installed between paired chromosomes, which is composed of two substructures, that is, lateral elements (LEs, referred to as chromosome axis before homologous synapsis) and central region (CR). The LE is a proteinaceous structure assembled along the entire length of sister chromatids upon meiotic entry. It comprises of a couple of axial proteins in plants including cohesin complexes, two “axis core” proteins ASY3 (homolog of Red1 in yeast, SYCP2 in mammals) and ASY4 (homolog of SYCP3 in mammals, no homolog identified in yeast), and the HORMA domain‐containing protein ASY1 (homolog of Hop1 in yeast, HORMAD1/2 in mammals) [9, 10, 11, 12, 13, 14, 15, 16].

In contrast, the CR starts to be installed between two coaligned LEs only at zygotene, completes when homologous chromosomes fully pair at pachytene, and disassembles during diplotene and diakinesis across all sexually reproducing eukaryotes studied including yeast, mammals, and plants [1, 2, 4]. It comprises of the central element (CE) proteins localizing in the center of CR/SC, and the interdigitating transverse filaments (TFs) that spans the CR and connects the LEs and CE [4]. In plants, while the core CR components, including TF protein ZYP1 and CE proteins like SCEP1/SCEP2, have been identified, the precise mechanisms governing the SC assembly, particularly within the CR, remain poorly understood. Recent studies established that ZYP1 polymerization into SC filaments requires CE proteins SCEP1 and SCEP2 that forming a heterodimer, but the molecular link between TFs (ZYP1) and the CE (SCEP1/SCEP2) remains elusive [17]. In addition, the importance of SC on recombination control in polyploid species is also less investigated.

In *Arabidopsis thaliana* (*A. thaliana*), the polymerization of ZYP1 into functional SC filaments fails in *scep1* or *scep2* mutants, and vice versa [17]. However, no direct interaction was found between ZYP1 and SCEP1/2 [17]. This likely suggests the existence of an unidentified architectural scaffold protein that bridges ZYP1 and SCEP1‐SCEP2 heterodimer to nucleate CR assembly. Furthermore, while SC disruption increases CO frequency, abolishes CO interference, and eliminates heterochiasmy (sex‐specific CO differences) in *A. thaliana* [17, 18, 19, 20, 21, 22], the extent to which this machinery modulates recombination in polyploids is largely unknown.

Here, we identify ZSL (for ZYP1‐SCEP1/2 Linker), a homolog of the recently identified *A. thaliana* SCEP3 [19, 21], as the missing molecular scaffold that orchestrates the CR assembly of the SC in *B. napus*. Combining transcriptomic profiling, cytogenetics, super‐resolution microscopy, and biochemical analyses, we demonstrate that ZSL is essential for the polymerization of the TF protein ZYP1 and the CE proteins SCEP1/2 into mature SC structures. Crucially, we establish that ZSL localizes in the center of the SC and serves as a central molecular adaptor in SC maturation, through a direct interaction with both ZYP1 and SCEP1/2 heterodimer. We further uncover the impact of ZSL deficiency on the genome‐wide CO formation and distribution in *B. napus*, revealing a conserved CO‐limiting role of the SC in polyploid meiosis.

## 2 Results

### 2.1 Identification of Genes Highly Expressed During Male Meiosis of *B. napus*

**FIGURE 1:**
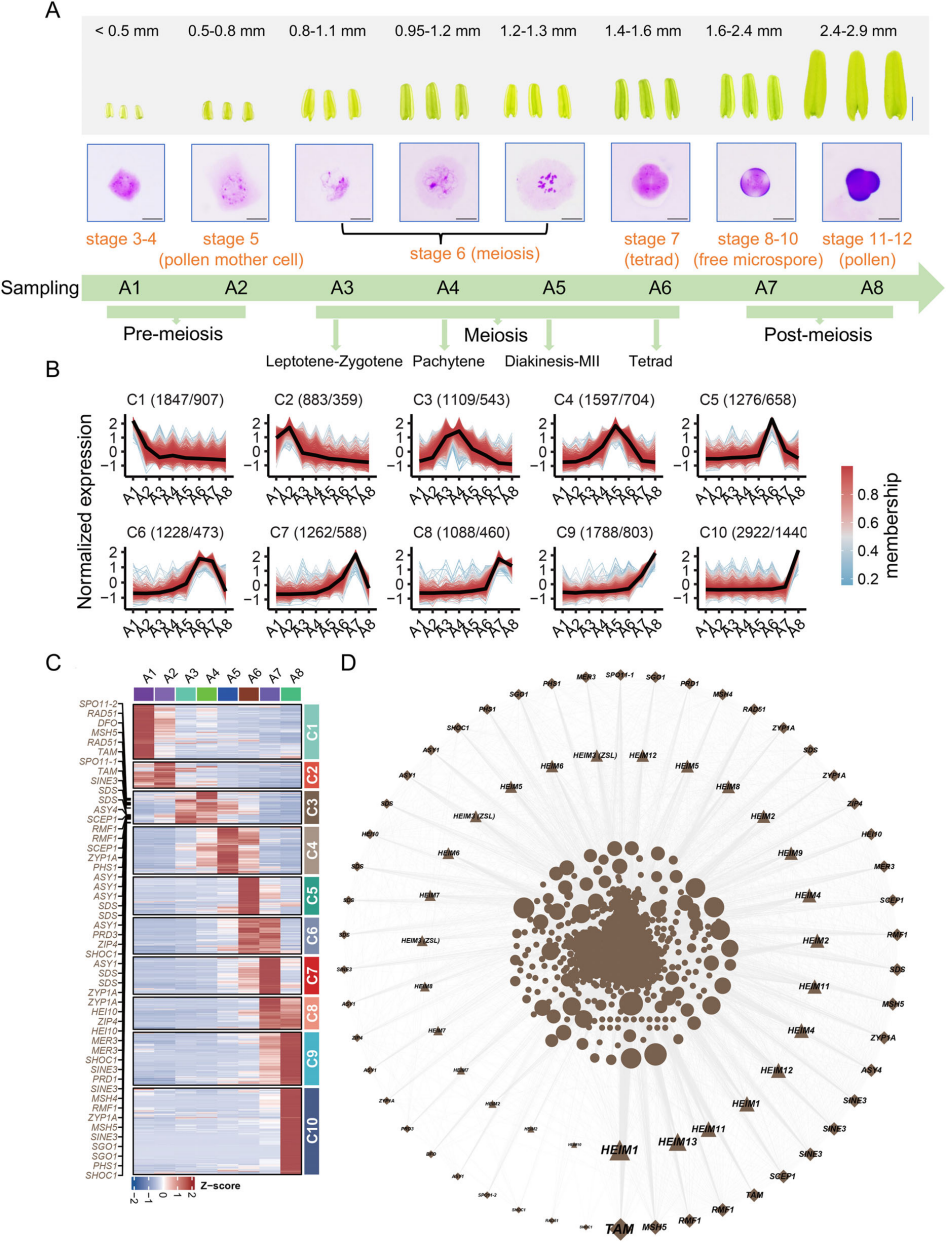
Temporal transcriptome analysis for anthers collected at different developmental stages in *Brassica napus*. (A) Diagram of anther collections (A1–A8) at eight developmental stages covering early anther development and pre‐meiosis (stage 3–5), meiosis (stage 6 and 7) to post‐meiosis (stage 8–12). The samples during meiosis were collected separately at four sub‐stages (leptotene/zygotene, pachytene, diakinesis to metaphase II, and tetrad stage). Each sample has three biological replicates. The upper panel shows representative images of dissected anthers and anther lengths. The lower panel shows images of archesporial cell, meiocyte, tetrad, microspore, and pollen stained by Carbol fuchsin solution. (B) Identification and clustering analysis for the 15000 *B. napus* genes varied significantly in expression level among eight anther development stages. Each cluster represent a distinct expression pattern. For each cluster, one line represents an individual gene. Light blue lines indicate genes with low values of membership and red lines indicate genes with high values of membership. The number of genes in each cluster and the corresponding number of homologues in *A. thaliana* are labeled on top of each graph. (C) Heatmaps of gene expression in 10 clusters shown in (B). Many known meiotic genes were enriched in cluster C3. (D) Correlation network of genes from the C3 cluster (Pearson's coefficients > 0.95 or < −0.95, *p*‐value < 0.05). Diamonds highlight known meiotic genes, triangles indicate the 13 selected candidate genes (*HEIM1*–*HEIM13*), and circles represent other genes in this cluster. The size of the shape depicts the connection degree.

Recent studies in *A. thaliana* have identified several new meiotic players based on the transcriptomic analysis searching the genes showing a significant high expression in flower buds undergoing meiosis as the majority of known meiotic genes, suggesting an effective approach for studying genes involved in meiosis [17, 23]. To identify potential players in plant meiosis, especially the polyploid meiosis, we collected stamen anthers of *B. napus* variety *Westar* covering different developmental stages from stages 3 to 4 harboring archesporial cells (<0.5 mm) (named as sample A1), stage 5 forming pollen mother cell (0.5–0.8 mm) (sample A2), stage 6 carrying out meiosis (0.8–1.3 mm) (sample A3–A5 at three substages of meiosis), stage 7 completing meiosis to form tetrad (1.4–1.6 mm) (sample A6), stages 8–10 containing free microspores (1.6–2.4 mm) (sample A7), to stages 11–12 producing bicellular and tricellular pollens (>2.4 mm) (sample A8) (Figure 1A) (see the Experimental Section for the collecting details). The meiotic anthers (stage 6) were further divided into anthers at leptotene/zygotene (sample A3), pachytene (sample A4), and diakinesis to metaphase II (sample A5) according to the chromosome behaviors under Carbol fuchsin staining.

These eight anther samples (each having three biological replicates) were subjected to the transcriptome sequencing using the illumina platform, producing a total of 1.098 billion high‐quality reads (Table S1). These reads were aligned to the *B. napus* variety *ZS11.v0* reference genome [24], with an average mapping rate of 89.53% (Table S1). The mapped reads were used to calculate transcripts per million mapped reads (TPM). The Pearson correlation coefficients (*R* ^2^) between replicates exceeded 0.90 (Figure S1A; Table S2), and the principal component analysis (PCA) showed that replicates of the same sample clustered together (Figure S1B), indicating a good consistency of our sampling. Thus, the mean TPM value from all replicates was taken as the gene expression level.

Next, we identified the 15,000 genes with the highest coefficients of variation (CV) in expression and clustered them using the fuzzy c‐means algorithm [25]. Ten clusters (C1–C10) were yielded, each representing a distinct gene expression pattern across different anther developmental stages, which provides a useful transcriptome variation map for studying *B. napus* male reproductive development (Figure 1B). We focused particularly on the genes highly expressed during early meiosis (stage 6: samples A3, A4, and A5). Interestingly, genes in cluster C3 exhibited high expression in samples A3 and A4 during leptotene to pachytene stages, while genes from cluster C4 showed high expression in sample A5 during diakinesis to metaphase II. Strikingly, cluster C3 was enriched for many known genes involved in key events of early meiosis including genes encoding DSB formation and repair factors (*SPO11‐1*, *SPO11‐2*, *DFO*, *PRD1*, *PRD3*, *RAD51*, *SDS*, and *PHS1*), components of the synaptonemal complex (*ASY1*, *ASY4*, *ZYP1A*, and *SCEP1*), factors required for class I CO formation (*ZIP4*, *HEI10*, *SHOC1*, *MSH4*, *MSH5*, and *MER3*), and other processes (SINE3, TAM, RMF1, and SGO1). Consistently, genes involved in late meiosis I or meiosis II (for cohesin complex protection, cell cycle transition, and/or microtubule organization) were enriched in cluster C4, for example, *TDM1*, *DUET*, *PANS1*, *OSD1*, and *PS1* (Figure 1C and Table S4).

### 2.2 HEIM3/ZSL Is Essential for Plant Fertility and Meiosis in *B. napus*

To identify the potential new players involved in early meiosis, we focused on cluster C3 that involves 1,109 genes (correspond to 543 homologous genes). We calculated Pearson correlation coefficients to construct a gene co‐expression network among these 1,109 genes (see the Experimental Section, Table S3). The known meiotic genes showed mostly moderate‐to‐high connection degree (≈50–206) with other genes (Figure 1D) (Table S4). In addition to the connectivity, we also consider gene copy number since previous studies showed that meiotic gene duplicates in angiosperms including *B. napus* experienced a rapid loss during evolution following whole genome duplication [26, 27]. Therefore, based on the following three criteria, that is, connection degree, gene copy number ≤ 4, and unknown function, we selected 13 candidate genes from cluster C3 to verify their potential function in meiosis. We named these genes of unknown function as High Expression in Meiosis 1–13 (HEIM1 to HEIM13) (Figure 1D, Table S4).

**FIGURE 2:**
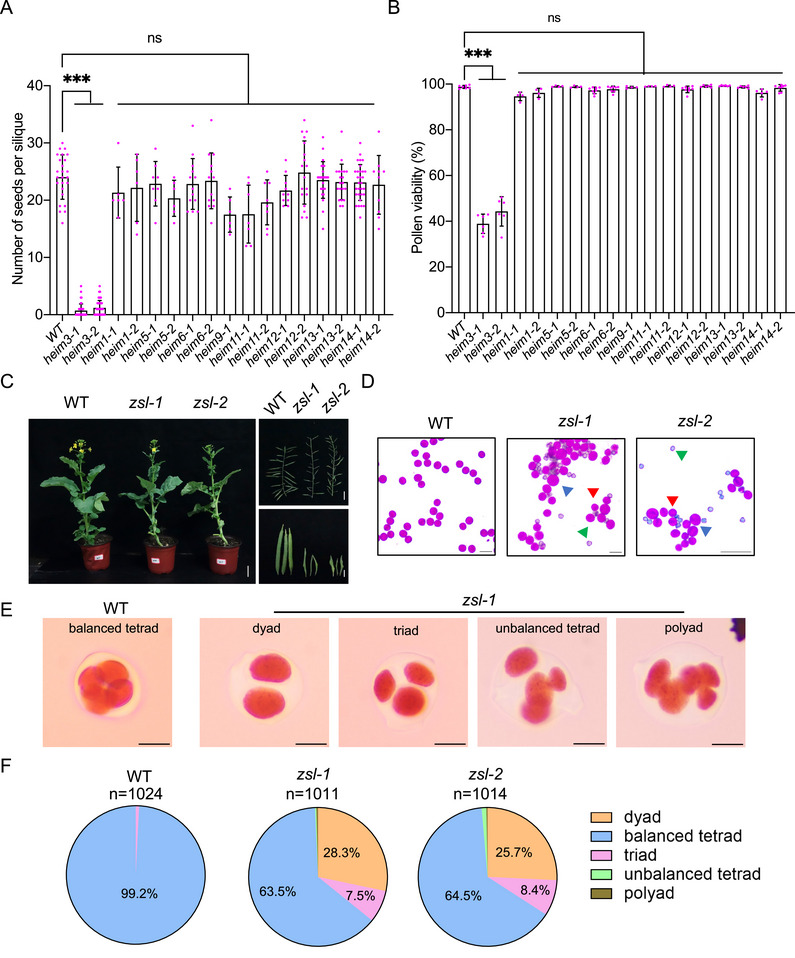
Mutation of *heim3*/*zsl* results in reduced fertility and defective meiosis in *Brassica napus*. (A) Statistical analysis of the number of seeds per silique in WT and *heim1* to *heim13* mutant plants. At least six siliques were dissected and counted for each genotype. Error bars indicate mean ± SD. Tukey's multiple comparison test, ***p < 0.001. (B) Statistical analysis of the pollen viability in WT and *heim1* to *heim13* mutant plants. At least 4000 pollen grains were counted from different flowers for each genotype. Error bars indicate mean ± SD. Tukey's multiple comparison test, ***p < 0.001. (C) Vegetative growth, main branches, and siliques of WT, *zsl‐1*, and *zsl‐2* mutant plants. Bars in panels of plant growth, main branch, and silique are 5, 5, and 1 cm. (D) Pollen staining of WT, *zsl‐1*, and *zsl‐2* mutant plants. Four plants were examined for each genotype. Blue arrowheads indicate pollen grains with larger size, red for normal size, and green for dead pollens. Bars: 100 µm. (E) Staining of male meiotic products at tetrad stage in WT, *zsl‐1*, and *zsl‐2* mutant plants. Bars: 5 µm. (F) Pie charts depicting the proportion of balanced tetrad, unbalanced tetrad, polyad, triad, and dyad WT, *zsl‐1*, and *zsl‐2* mutant plants.

To verify whether these 13 candidate genes play a role in meiosis, we sought to generate their mutants using CRISPR‐Cas9 gene editing approach (Figure S2). As a result, homozygous mutants without Cas9 were successfully generated for 8 out of 13 genes, carrying either insertions or deletions that cause premature translation termination, whereas the gene editing was failed for the rest 5 genes (Figure S2). All of these mutants exhibited normal vegetative growth (Figure S3). At reproductive stage, *heim3* homozygous mutants showed strongly reduced fertility, while the homozygous mutants of rest 7 genes had normal seed setting rates (Figure 2A,C). Consistently, pollen staining by Peterson solution showed that only *heim3* showed reduced viability with varied sizes (Figure 2B,D). Further tetrad analysis showed a severe disruption of meiotic products in *heim3* (36.5% abnormal tetrads in *heim3‐1*, 35.5% in *heim3‐2* vs 0.8% in wildtype) (Figure 2E,F). These results suggest that HEIM3 plays an important role in meiosis. Hereafter, we renamed HEIM3 as ZSL (for ZYP1‐SCEP1/2 Linker) based on its molecular role (see details below). *ZSL* has four copies in *B. napus* genome including *BnaA01.ZSL* (BnaA01G0015100WE), *BnaA03.ZSL* (BnaA03G0455300WE), *BnaC01.ZSL* (BnaC01G0088700WE), *BnaC06.ZSL* (BnaC06G0182500WE) and two copies exist in genomes of its progenitor species *B. rapa* and *B. oleracea*, suggesting ZSL from both the A and C sub‐genomes were retained after the allo‐hybridization to form *B. napus*. PSI‐BLAST analysis revealed that ZSL homologues are widely present in angiosperms and also exists in ferns (Figure S4).

### 2.3 ZSL Is Required for Homologous Synapsis

**FIGURE 3:**
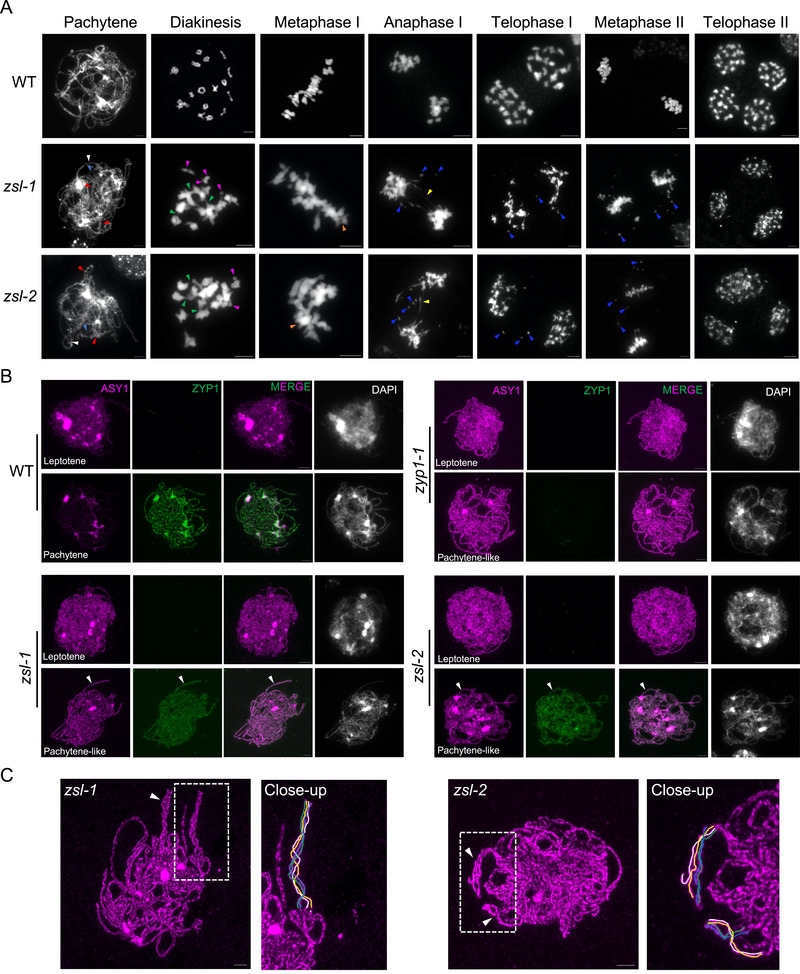
Analysis of meiotic chromosome behavior in wildtype (*Westar*) and *zsl* mutants in *Brassica napus*. (A) Chromosome spread analysis of male meiosis in WT, *zsl‐1*, and *zsl‐2* mutant plants. Flower buds from at least ten plants were collected and analyzed for each genotype. White arrowheads indicate large gaps between coaligned chromosomes, light blue arrowheads indicate single chromosomes, red arrowheads indicate regions of multivalent pairing, green arrowheads indicate chromosome entanglements, magenta arrowheads indicate univalent, brown arrowheads indicate multivalent‐like configurations, yellow arrowheads indicate chromosome bridges, and blue arrowheads indicate chromosome fragments. Bars: 5 µm. (B) Co‐immunostaining of ASY1 and ZYP1 at leptotene and pachytene (or pachytene‐like) stages in male meiocytes of WT, *zyp1‐1*, *zsl‐1*, and *zsl‐2* mutant plants. White arrowheads indicate regions where ZYP1 is loaded yet not properly assembled into functional SC in *zsl* mutants, exhibiting a wider diffuse signal compared to the tightly co‐aligned linear structures in WT. Noting that no ZYP1 signal was detected in *zyp1* mutants. Bars: 5 µm. (C) Multivalent pairing/co‐alignment with pairing partner switches in *zsl* mutants. White arrowheads indicate regions exhibiting multivalent associations. Bars: 5 µm.

To further understand the detailed impact of the mutation of *zsl* on meiosis in *B. napus*, we performed chromosome spread analysis in male meiocytes. In the wildtype, despite the presence of closely related homoeologous chromosomes, homologous chromosomes pair and co‐align at pachytene, subsequently forming 19 discernible bivalents that are linked by the chiasmata (COs in between) at diakinesis (Figure 3A). All bivalents are moved to and align on the equatorial plane at metaphase I, and non‐sister chromatids from each bivalent are separated equally at anaphase I. During the second meiotic division, sister chromatids segregated equally into opposite poles, producing four haploid gametes at tetrad stage (Figure 3A).

In *zsl* mutants, no obvious differences were found at early meiosis. However, at pachytene‐like stage, no typical pachytene‐like cells were observed in *zsl* mutants, and instead, while many chromosome regions are coaligned, some obvious alterations were seen, including single chromosome threads (light blue arrowheads, *n* = 16 out of 46 cells in *zsl‐1*, 17 out of 25 in *zsl‐2*), large gaps between paired chromosomes (white arrowheads, *n* = 27 out of 46 cells in *zsl‐1*, 9 out of 25 in *zsl‐2*), and even coalignments of multiple chromosomes (red arrowheads, *n* = 15 out of 46 cells in *zsl‐1*, 11 out of 25 in *zsl‐2*), suggesting that chromosome pairing and synapsis is likely compromised in the absence of ZSL (Figure 3A). Unlike the formation of 19 bivalents in wildtype at diakinesis which migrate to the equator plane at metaphase I, we observed several abnormalities in *zsl* mutants during diakinesis to metaphase I, that is, the presence of univalents in addition to bivalents (*n* = 22 out of 93 cells in *zsl‐1*, 18 out of 100 in *zsl‐2*), the abnormal chromosome entanglements/connections (*n* = 54 out of 93 cells in *zsl‐1*, 63 out of 100 in *zsl‐2*), and some multivalent‐like configurations (*n* = 6 out of 93 cells in *zsl‐1*, 6 out of 100 in *zsl‐2*) (Figure 3A). At anaphase I, accompanying by the unequal separation, some chromosome bridges and fragments were present in all meiocytes (*n* = 24 cells for *zsl‐1*, 26 for *zsl‐2*), likely due to the ectopic entanglements/connections seen at diakinesis/metaphase I and/or an inefficient DSB repair. These fragments and entanglements continue to be seen during meiosis II, producing unbalanced gametes at tetrad stage (Figure 3A). These observations are a reminiscence of the meiotic defects in *B. napus zyp1* mutants where the defects are even more severe [8]. Nevertheless, we found the duration of meiotic progression for prophase I and meiosis II is not dramatically affected by the loss of ZSL according to the correlation between anther sizes and meiotic stages (Figure S5A).

Since the formation of double‐strand breaks (DSBs) play important role in chromosome pairing, synapsis, and recombination, we first confirmed that the DSB formation is largely normal in *zsl* mutants by performing immunostaining for DMC1 that localizes to DSB sites, suggesting that other downstream factors are responsible for the meiotic defects seen in *zsl* mutants (Figure S6). Next, we performed co‐immunostaining to study the pairing and synapsis using antibodies against ASY1 and ZYP1. In wildtype, ASY1 is assembled on the axis at leptotene, and gets progressively removed from the synapsed regions as indicated by the installation of ZYP1 from zygotene onwards, showing a complete removal at pachytene except for the heterochromatic regions including centromeres when the SC/ZYP1 is fully installed (Figure 3B). In *zsl* mutants, ASY1 assembly is normal at early prophase I. However, we did not detect any obvious removal of ASY1 from the paired and coaligned axes till pachytene‐like stage, and at the same time, no wildtype‐like ZYP1 assembly, even not the short SC patches, was formed, indicating that the SC assembly is disrupted in the absence of ZSL (Figure 3B). Notably, ZYP1 exhibits a fuzzy and diffused pattern associated with chromosomes in *zsl* (Figure 3B and see below). In addition, several single ASY1 threads were detected, indicating that some chromosome regions are not successfully co‐aligned in *zsl* mutants, in accordance with the DAPI‐stained spreads. In some pachytene‐like cells (31% in *zsl‐1*, *n* = 48; 33% in *zsl‐2*, *n* = 43), we even observed the association of multiple chromosomes, which involved pairing partner switches (Figure 3C). All these defects observed in *zsl* mutants are similar to that in *B. napus zyp1* mutants where the SC assembly is also completely abolished, supporting the role of the SC in inhibiting the multivalent interactions in polyploids [7, 8].

### 2.4 ZSL Localizes on the Synaptonemal Complex

**FIGURE 4:**
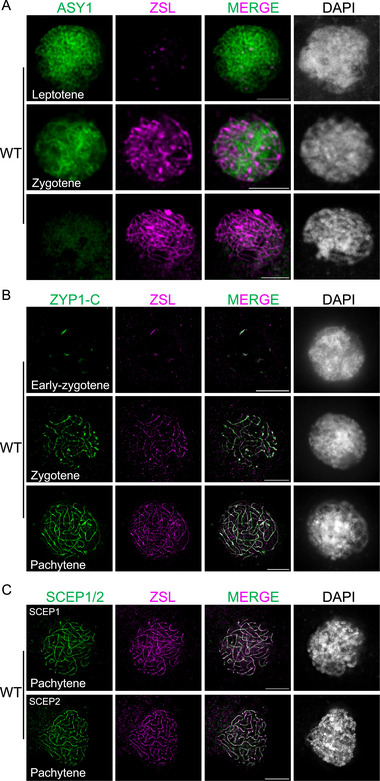
ZSL localizes on the SC. (A) Co‐immunolocalization of ZSL and ASY1 in male meiocytes of wildtype *B. napus* (*Westar*) at different prophase I stages. Bars: 5 µm. (B) Co‐immunolocalization of ZSL and ZYP1 in male meiocytes of wildtype *B. napus* (*Westar*) at different prophase I stages. ZYP1 was detected using the ZYP1‐C antibody against its C‐terminal region. Bars: 5 µm. (C) Co‐immunolocalization of ZSL and SCEP1/2 in male meiocytes of wildtype *B. napus* (*Westar*) at pachytene. Bars: 5 µm.

The meiotic similarity between *zsl* and *zyp1* mutants led us to wonder whether ZSL belongs to a component of the SC. To answer this question, we performed co‐immunostaining on the male meiocytes of wildtype using a newly generated antibody against ZSL and the antibody against ASY1 for staging. The ZSL antibody was raised against the N‐terminal part of BnaC06.ZSL (1‐225 aa), which recognize all ZSL copies from the A and C sub‐genomes (Figure S5B). Our observations using the confocal microscope show that at leptotene when ASY1 forms linear thread‐like signals, only a few dotty or short stretch‐like ZSL signals presents on ASY1‐labelled axes (Figure 4A). At zygotene, ZSL forms thread‐like signals along chromosomes, which only localizes on the synapsed regions indicated by the partial removal of ASY1 proteins. At pachytene, linear ZSL signals were detected to localize along all synapsed chromosomes where ASY1 is largely removed (Figure 4A). Notably, no ZSL signal was detected in pachytene‐like cells of *zsl‐1* and *zsl‐2* mutants (Figure S5C). These results indicate that ZSL specifically localizes onto the synapsed chromosomes. We further confirmed this conclusion through the co‐immunolocalization of ZSL with ZYP1 (antibody against its C‐terminal 503‐876 aa) or the CE proteins SCEP1/2 at pachytene (Figure 4B,C).

### 2.5 ZSL Is a Component of the Central Element of the SC

**FIGURE 5:**
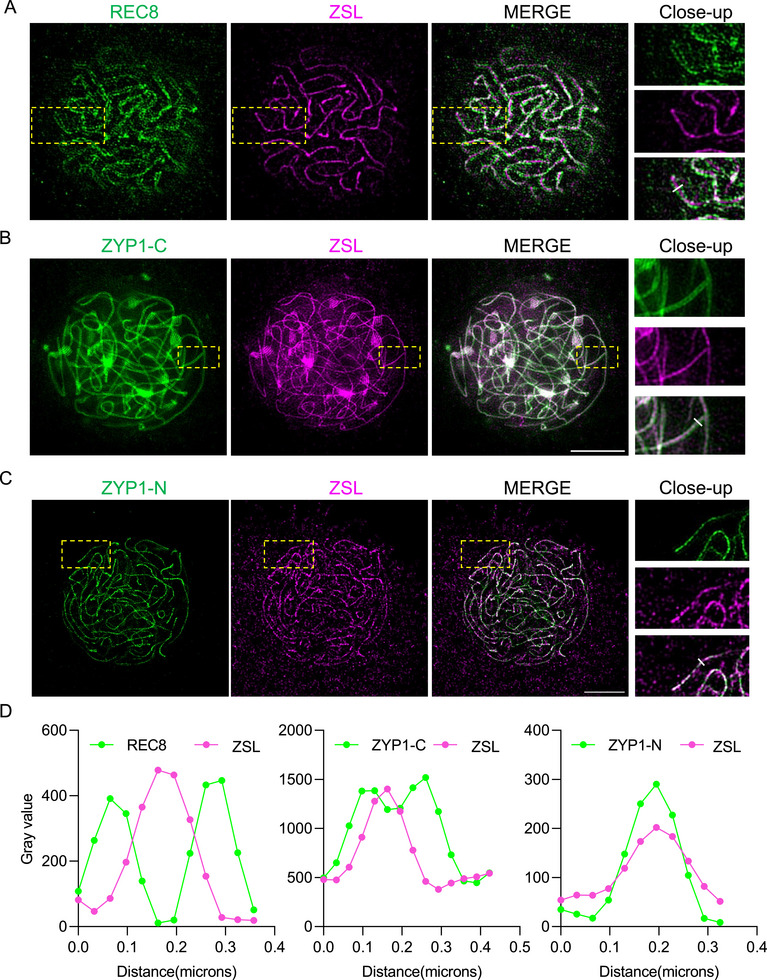
ZSL localizes at the center of the SC. (A) Co‐immunolocalization of ZSL and REC8 in male meiocytes of wildtype *B. napus* (*Westar*) at pachytene. Bars: 5 µm. (B) Co‐immunolocalization of ZSL and the C‐terminal region of ZYP1 (ZYP1‐C) in male meiocytes of wildtype *B. napus* (*Westar*) at pachytene. (C) Co‐immunolocalization of ZSL and the N‐terminal region of ZYP1 (ZYP1‐N) in male meiocytes of wildtype *B. napus* (*Westar*) at pachytene. (D) signal distribution profiles of ZSL with REC8, ZYP1‐C, or ZYP1‐N, respectively, as shown in (A–C). The region used for analysis is indicated by white lines in the close‐up panels. All images were captured by super resolution SIM microscope. Bars: 5 µm.

To investigate the detailed localization of ZSL on the SC, we co‐immunostained ZSL with antibodies against the lateral element component REC8 and two antibodies against the N‐ or C‐ terminal parts of ZYP1 that recognize all ZYP1 copies from A and C sub‐genomes, and captured the images by the super‐resolution structured illumination microscopy (SIM). At pachytene, REC8 forms two parallel axis threads separated by a distance of 200 ± 12 nm (measured between the signal peaks), and ZSL appears as a single line that localizes between two REC8 lines (Figure 5A,D), suggesting that ZSL localizes at the central region of the SC. Thus, we further compared the localization of ZSL with the N‐ (ZYP1‐N) or C (ZYP1‐C)‐terminal part of ZYP1 that spans the central region of the SC with ZYP1‐N integrated into the central element of the SC. As expected, staining with the antibody raised against C‐terminal part of BnaA07. ZYP1B (503‐876 aa), which recognize all four ZYP1 copies from A and C sub‐genomes, reveal two parallel lines separated by a much closer distance (124 ± 12 nm) compared to REC8 lines. Interestingly, we found that ZSL single line presents even in the middle of the two lines of ZYP1‐C (Figure 5B,D). In contrast, ZSL co‐localizes with ZYP1‐N (antibody raised against 1‐390 aa of BnaA07. ZYP1B) that shows a single line running in the middle of two REC8 lines (Figure 5C,D). Together with the necessity of ZSL for the SC/ZYP1 assembly, these results suggest that ZSL is a component of the central element of the SC.

### 2.6 ZSL and SCEP1 Is Not Required for the Recruitment of ZYP1 to Co‐aligned Chromosomes

In *zsl* mutants, although the linear thread‐like ZYP1/SC assembly is completely failed at pachytene‐like stage, we found that many diffused ZYP1 proteins are associated with the co‐aligned axes, despite having a relatively weaker signal intensity (Figure 2B), suggesting that the recruitment of ZYP1 to the axes/chromosomes is largely not impacted by the loss of ZSL. The fact that no ZYP1 signal could be detected in *B. napus zyp1* mutants, supports the specificity of ZYP1 signal in *zsl* mutants (Figure 2B) [8]. This result imply that the central element is important for the polymerization of ZYP1 to form the SC, but not for its chromosome recruitment.

**FIGURE 6:**
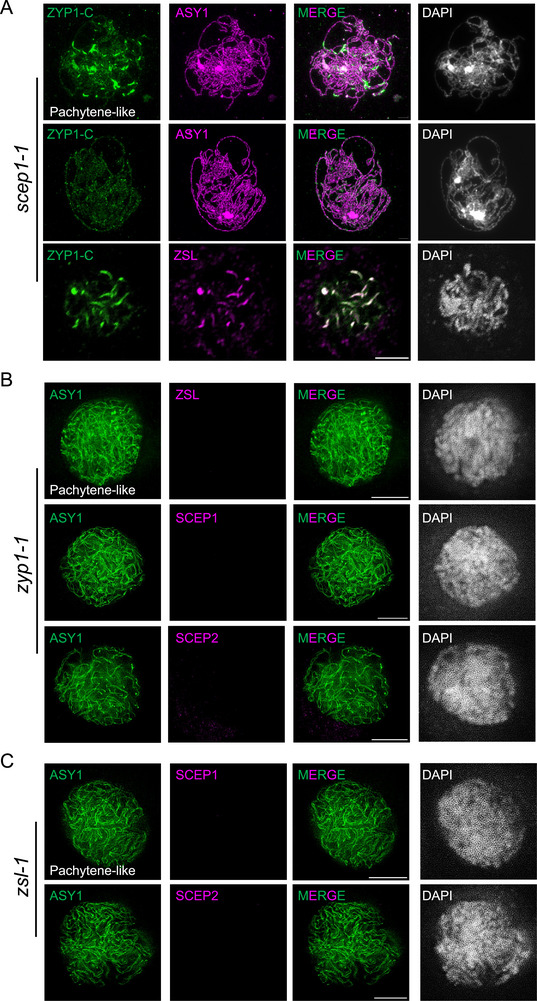
Localization interdependency among ZSL, ZYP1, and SCEP1/2. (A) Co‐immunolocalization of ZYP1 with ASY1 or ZSL with ASY1 in male meiocytes of *B. napus scep1‐1* mutants at pachytene‐like stage. (B) Co‐immunolocalization of ZSL with ASY1, SCEP1 with ASY1, or SCEP2 with ASY1 in male meiocytes of *B. napus zyp1‐1* mutants at pachytene‐like stage. (C) Co‐immunolocalization of SCEP1 with ASY1 or SCEP2 with ASY1 in male meiocytes of *B. napus zsl1‐1* mutants at pachytene‐like stage. All images were acquired by confocal microscope. Bars: 5 µm.

To further confirm this conclusion, we sought to check the localization of ZYP1 in mutants of another two recently identified central element components SCEP1 and SCEP2, two small coiled‐coil proteins that forms a heterodimer [17]. We identified two and three homolog copies for SCEP1 and SCEP2 in the genome of *B. napus* variety *Westar*, respectively and generated *scep1* null mutants via CRISPR‐Cas9 gene editing approach (Figure S7). Co‐immunostaining of ASY1 and ZYP1 in *scep1* mutants showed that the wildtype‐like ZYP1 polymerization is strongly compromised, coinciding with the defective removal of ASY1 from co‐aligned chromosomes (Figure 6A). While some bright ZYP1 short patches were detected in a portion of pachytene‐like cells of *scep1* mutants (53%, *n* = 57) (notably, these patches are unlike the short functional SC segments, but more like the ectopic ZYP1 protein accumulations since ASY1 cannot get removed from these ZYP1 accumulated patches), many diffused ZYP1 signals, though reduced in intensity, were associated with the paired chromosomes in all cells (*n* = 57) (Figure 6A), similar to that in *zsl* mutants, substantiating the crucial role of the central element in faithful polymerization of ZYP1/SC along paired axes, but less important for ZYP1 recruitment per se.

Next, we asked the role of the transverse filaments/ZYP1 in localization of central element components, ZSL and SCEPs. Immunostainings for ZSL, SCEP1, and SCEP2 showed that all of them were completely absent in male meiocytes of *zyp1* mutants (Figure 6B; Figure S5D), suggesting that ZYP1/transverse filament is required for the loading of central element components in *B. napus*, consistent with previous studies including *A. thaliana* [17, 28, 29, 30].

We then wondered the mutual impact between ZSL and SCEPs. We found that ZSL forms only few short stretches in *scep1* mutants, which co‐localizes with ZYP1 (Figure 6A), suggesting that SCEP1 is required for the normal assembly of ZSL. In *zsl* mutants, we did not detect any SCEP1 and SCEP2 signals, suggesting that the loading and assembly of SCEP1 is strictly dependent on ZSL (Figure 6C).

### 2.7 ZSL Bridges ZYP1 and SCEPs to Form the SC Central Region

**FIGURE 7:**
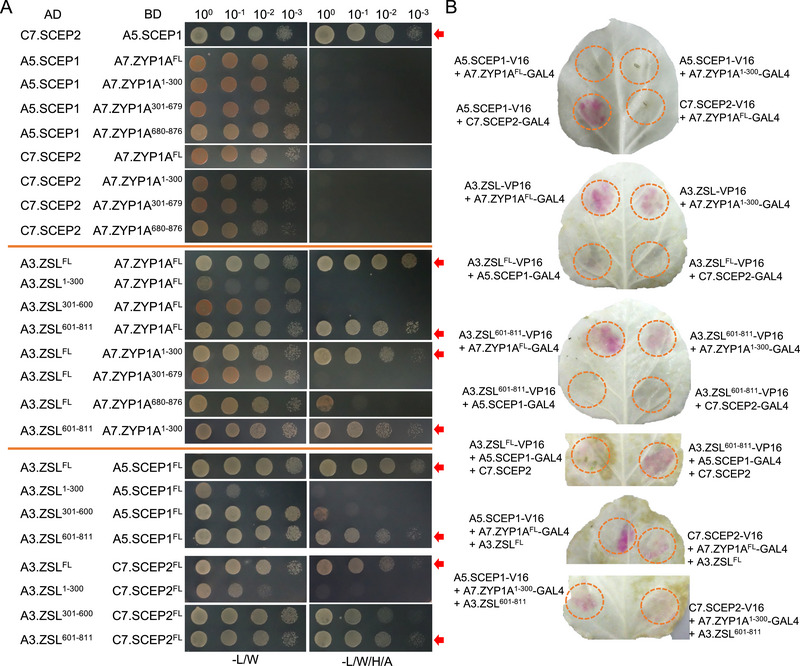
ZSL interacts with both ZYP1 and SCEP1/2 heterodimer in *Brassica napus*. (A) Yeast two‐hybrid assay testing for interaction of ZYP1 with SCEP1/2, ZSL with ZYP1, or ZSL with SCEP1/2. For each protein, one of the homologous copies from either A or C sub‐genome was used. In addition to the full‐length proteins, three truncations were tested for ZYP1 (1‐300, 301‐679, 680‐876 aa) and ZSL (1‐300, 301‐600, 601‐811 aa), respectively. (B) Split GAL4 RUBY assays for interaction of ZYP1 with SCEP1/2, ZSL with ZYP1, or ZSL with SCEP1/2 in leaves of *N. benthamiana*. Red RUBY signal indicates positive interaction. In addition to the full‐length proteins, the truncations of ZYP1 (1‐300 aa) and ZSL (601‐811 aa) binding to other proteins in yeast two‐hybrid assay were tested in this system.

To understand how these CR proteins (ZYP1, SCEP1, SCEP2, and ZSL) assemble to form the SC central region, we tested their mutual interactions via yeast two‐hybrid (Y2H) assays. Since two or more functional redundant copies for each gene from the A and C sub‐genomes exist in *B. napus*, we cloned one of them for the Y2H. Consistent with previous reports in *A. thaliana* [17], while SCEP1 and SCEP2 interacts with each other in *B. napus*, we did not detect interaction between full length (FL) or truncated ZYP1A and FL‐SCEP1 or FL‐SCEP2 (Figure 7A). Interestingly, FL‐ZSL interacts with both FL‐ZYP1 and FL‐SCEP1 or FL‐SCEP2, as indicated by the growth of yeast cells on the stringent selection media (Figure 7A, see all controls in Figure S8). Further analyses revealed that the region responsible for the binding of ZYP1 to ZSL located at its N‐terminal part (1–300 aa) that co‐localizes with ZSL in the middle of the SC (Figures 7A and 3C). The region required for the binding of ZSL to ZYP1, SCEP1, and SCEP2 resided in its C‐terminal α‐helical domain (601–811 aa). Note that the yeast growth on the quadruple dropout selection medium for the combination of AD‐ZSL^301‐600^ and BD‐SCEP2‐FL is due to the autoactivation of BD‐SCEP2‐FL construct (Figure S8). We further confirm the interactions of ZSL with both ZYP1 and SCEP1/2 using the split GAL4 RUBY assays in leaves of *Nicotiana benthamiana* (red RUBY signal indicates positive interaction) [31] (Figure 7B, see all controls in Figure S8). No direct binding between ZYP1 and SCEP1 or SCEP2 was detected, consistent with the observation via Y2H (Figure 7A,B). Notably, in this split GAL4 RUBY system, the interaction between ZSL and SCEP1 or SCEP2 was relatively weak. However, this interaction become more robust when ZSL (especially for ZSL^601‐811^), SCEP1, and SCEP2 were co‐expressed (Figure 7B), suggesting that the heterodimer form of SCEP1 and SCEP2 strengthens their interaction with ZSL. Together, these results suggest that ZSL likely links ZYP1 and SCEP1/2 to form the CR of the SC in *B. napus*.

To confirm the linking role of ZSL in the CR assembly, we exploited the split GAL4 RUBY assays and verified the formation of ZYP1‐ZSL‐SCEP1/2 complex by expressing either only ZYP1 and SCEP1/2 or adding ZSL in addition in leaves of *N. benthamiana*. We found that the RUBY signal was produced only when ZSL was co‐expressed (Figure 7B). These results demonstrate that ZSL function as a linker in the CR protein assembly of the SC.

### 2.8 Absence of ZSL Increases CO Formation and Compromises CO Interference

In both *A. thaliana* and *B. napus*, the mutation of *zyp1* that disrupts the SC installation leads to an elevation of CO number and significantly attenuates the CO interference, suggesting the importance of the SC in regulating CO frequency and distribution [8, 18, 20]. To understand the impact of *zsl* mutation on recombination, we first performed the immunostaining of HEI10 at different stages of male meiosis. HEI10, a ring‐type E3 ubiquitin ligase, belongs to the ZMM group of proteins required for interference‐sensitive (also known as class I) CO formation. HEI10 exhibits a dynamic localization pattern during meiotic prophase I, which is conserved in many species including *B. napus* [32]. It initially forms numerous small foci along chromosomes at zygotene and early pachytene which are progressively integrate into large foci colocalizing with the MLH1‐maked class I CO sites at late pachytene, diplotene, and diakinesis [2, 32, 33, 34, 35].

**FIGURE 8:**
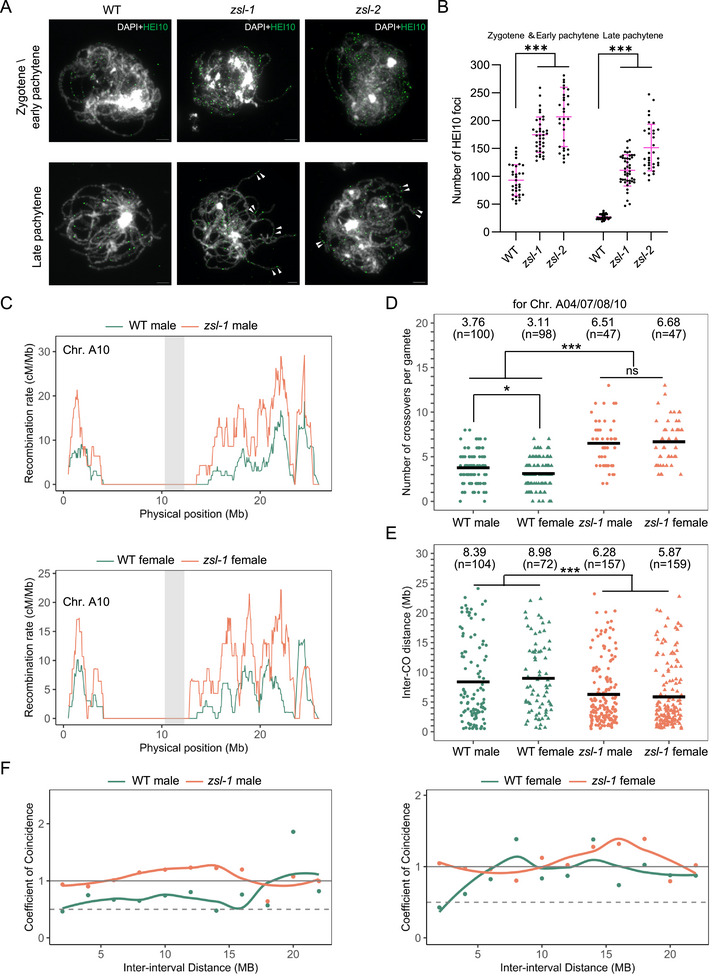
HEI10 foci and COs are increased in *zsl* mutants. (A) Immunolocalization of HEI10 at early prophase I (zygotene/early pachytene) and late pachytene in male meiocytes of WT, *zsl‐1*, and *zsl‐2* mutant plants. White arrowheads in the images of late pachytene indicate the closely localized HEI10 foci. Bars: 5 µm. (B) Quantification of the number of HEI10 foci at zygotene/early pachytene and late pachytene in male meiocytes of WT, zsl‐1, and zsl‐2 mutant plants. Error bars indicate mean ± SD. Tukey's multiple comparisons test, ***p < 0.001. (C) Distribution of COs along chromosome A10 in male and female WT and *zsl1‐1*. The A4, A7, and A8 chromosomes are presented in Figure S12. COs are analyzed following the whole‐genome re‐sequencing of the male and female backcrosses of F1 *Westar*/*J9707* hybrids. The centromeric regions are indicated by grey shading, respectively. The analysis was done with 1 Mb windows and 50 kb sliding steps. (D) Quantification of the number of COs per gamete for chromosome A4, A7, A8, and A10 in male and female WT and *zsl‐1* mutants. COs. Each dot indicates an individual BC1 plant. The mean value of each population is indicated at the top and *n* values represent the number of plants used for analysis. Two‐sided Mann–Whitney *U* test, *p < 0.05, ***p < 0.001. Note that since a given CO involves only two of the four chromatids of a recombined bivalent and that a gamete inherits only a single chromatid, the number of COs observed per gamete is on average one‐half of the total amount of COs per meiocyte. (E) Distribution of inter‐CO distances for adjacent COs in male and female WT and *zsl‐1* mutants. The mean value is indicated at the top and *n* values represent the number of observed double CO events. Tukey's multiple comparisons test, ***p < 0.001. (F) CoC analysis for chromosome A4, A7, A8, and A10 in male and female WT and *zsl‐1* mutants with a 2‐Mb distance between intervals.

In male meiocytes of wild‐type *B. napus*, a high number of HEI10 foci per cell at zygotene/early pachytene (93 ± 27, *n* = 33 cells) was detected, which was reduced to 27 ± 4 (*n* = 32 cells) at late pachytene (Figure 8A). In both *zsl‐1* and *zsl‐2* mutants, the number of HEI10 foci is significantly increased at zygotene/early pachytene‐like stage compared to the wildtype (174 ± 31, *n* = 38 cells in *zsl‐1* and 206 ± 52, *n* = 31 in *zsl‐2* vs 93 ± 27, *n* = 33 in wildtype, Tukey's multiple comparisons test, *p* < 0.001). A significant elevation in HEI10 foci number persists in *zsl* mutants at late pachytene‐like stage (111 ± 28, *n* = 46 cells in *zsl‐1* and 151 ± 41, *n* = 33 in *zsl‐2* vs 27 ± 4, *n* = 32 in wildtype, Tukey's multiple comparisons test, *p* < 0.001) (Figure 8B). While typically only one large HEI10 focus was observed in a long stretch of synapsed chromosomes at late pachytene in male meiocytes of wildtype, the closely localized HEI10 foci were prevalent in all late pachytene cells of *zsl* mutants (Figure 8A, white arrowheads). Notably, unlike in the wildtype, it is likely that not all HEI10 foci (especially the smaller ones) at late pachytene‐like stage in *zsl* mutants would become into mature COs. Nevertheless, these results indicate that the HEI10 coarsening process is affected by the absence of ZSL, suggesting a compromise of CO interference that leads to an increase in COs. These results resemble the situation in *B. napus zyp1* mutants reported recently [8].

Next, we sought to measure COs in genome‐wide level by applying the whole genome re‐sequencing, which requires another *zsl* mutation in a genetically distant variety of *B. napus*, for example, a winter‐type variety. However, the genetic transformation for the winter‐type *B. napus* is still challenging and rarely successful. Thus, we chose a new spring‐type variety *J9707* that can be transformed in our hands and generated a new *zsl* mutant allele using CRISPR‐Cas9 gene editing approach (*zsl‐3* in the variety of *J9707*) (Figure S9). Genome analysis showed that *J9707* is not highly divergent from *Westar*, as shown by the relatively low number and density of polymorphic markers across their genomes, whereas the A sub‐genomes showed a clearly higher level of polymorphism than C sub‐genomes (Figure S10). This likely allows us to analyze more precisely the CO formation in the A sub‐genome but not the C sub‐genome, as the low maker density reduces the accuracy of genotype calling (see method and below).

We created F1 null hybrids between the *Westar zsl‐1* and *J9707 zsl‐3* alleles. These F1 null hybrids and corresponding wild‐type hybrid controls were backcrossed as male or female to the wild‐type *Westar*, and the progenies obtained were subjected to whole‐genome sequencing for CO frequency and distribution analysis (the number of plants sequenced: wildtype male *n* = 100, wildtype female *n* = 98, *zsl* male *n* = 47, and *zsl* female *n* = 47). We initially tried to analyze the COs on all chromosomes in the wildtype using the available markers, and used the parameter that a double CO must be supported by at least 500 kb constant genomic interval (see method). As expected, this analysis revealed obvious errors, for example, the total CO number per meiosis (for twofolds of the number of COs per gamete) significantly deviated from the previous cytological observations (in average 79.44 in wild‐type males, 74.96 in wild‐type females vs 33.97 in wild‐type males based on cytological counting) [32] and no CO interference (manifested by the Coefficient of Coincidence (CoC) analysis calculating the ratio between the observed frequency and expected frequency between two intervals) was detected when considering all chromosomes (Figure S11A,B). Although the number of COs on most of the chromosomes are deviated from cytological observations [32], it showed a reasonable CO formation for chromosome A04, A07, A08, and A10 that have higher polymorphic maker density (in average ≤ 2 COs per meiosis), consistent with the cytological observation (Figure S11C). Furthermore, the CoC cure for Chr. A04/07/08/10 revealed the exist of CO interference at short distances (CoC below 1) in wildtype, which disappears at larger distances (CoC close to 1 or above) (Figure 8F).

Therefore, we compared the CO frequency and distribution on Chr. A04/07/08/10 in wildtype and *zsl* mutants. In both male and female *zsl*, CO frequencies increased along chromosome arms, especially towards chromosome ends and even within regions close to centromeres (Figure 8C; Figure S12). In male meiosis, the average number of COs per gamete for Chr. A04/07/08/10 was increased from 3.76 ± 1.82 (mean ± SD) in wildtype to 6.51 ± 2.62 in *zsl* mutants (Figure 8D), thus an ∼100% increase. In female wildtype, the average number of COs for these four chromosomes was 3.11 ± 1.68 per gamete, which is slightly, yet significantly lower (∼20% less) than that in male wildtype (Figure 8D). This difference in CO frequency of male and female in *B. napus* is not as marked as that in *A. thaliana* (∼1.5–2‐fold in male vs female), a phenomenon called heterochiasmy [18, 36]. Remarkably, the number of COs in female *zsl1* Chr. A04/07/08/10 was more than double compared to wildtype (6.68 ± 2.54) and reached the same level as observed in male *zsl1* (6.51 ± 2.62) (Figure 8D). Thus, the absence of ZSL increases COs in both male and female meiosis and eliminates the difference between two sexes in *B. napus*.

We further compared the distance between two COs that occurred on the same chromosome. We found that in average more double COs were formed in *zsl* mutants (157 for 47 *zsl* male individuals and 159 for 47 *zsl* female individuals vs 104 for 100 wild‐type male individuals and 72 for 98 wild‐type female individuals), consistent with the increased CO formation in the absence of ZSL. The average distance between adjacent COs is significantly decreased in *zsl* compared to the wildtype, indicating the attenuation of CO interference (Figure 8E). The CoC cures showed that CO interference was abolished in *zsl* mutants (Figure 8F). Collectively, these results suggest that CO numbers are increased genome‐wide in the absence of ZSL, and support a conserved role of the SC in limiting CO formation by strengthening the interference.

## 3 Discussion

Our study identifies ZSL as a central region scaffold of the SC assembly in the allotetraploid crop *B. napus*, and reveals its essential role in ensuring homologous synapsis, regulating CO number and distribution, and maintaining meiotic stability in polyploid meiosis. We show that ZSL directly interacts with both the TF protein ZYP1 and the CE heterodimer SCEP1/2, thereby bridging/facilitating these two SC substructures to enable CR polymerization. This work closes a critical gap in our understanding of the mechanism of SC assembly in plants by identifying the missing molecular link between ZYP1 and the SCEP1/2. Furthermore, we reveal new insights into the role of the SC in recombination control in polyploid meiosis, providing potential gene target to modulate CO frequency for breeding.

### 3.1 A Distinct Hierarchical Assembly of SC in *B. napus* Compared to *A. thaliana*

**FIGURE 9:**
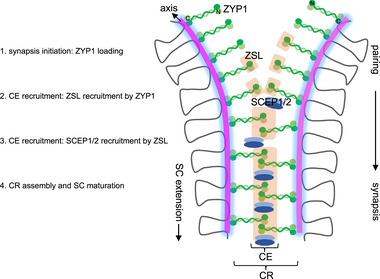
Model for ZSL‐facilitated synaptonemal complex maturation in *B. napus*. Synaptonemal complex (SC) assembly begins with the loading of the transverse filament (TF) protein ZYP1 between paired chromosome axes/lateral elements (LEs), a process independent of central element (CE) components. ZYP1 subsequently recruits the CE protein ZSL, which acts as an adaptor to recruit additional CE proteins, such as the SCEP1–SCEP2 heterodimer. The incorporation of ZSL, SCEP1–SCEP2, and other CE proteins completes the assembly of the central region (CR) between LEs, thereby promoting and stabilizing SC maturation and extension along the entire chromosome length.

In *B. napus*, neither ZYP1 nor SCEP1/2 is able to form wildtype‐like tight and linear structures in the absence of ZSL although homologous chromosomes are largely co‐aligned, suggesting the essential role of ZSL in SC assembly per se. Interestingly, although the polymerization of ZYP1 into functional SC filaments requires both ZSL and SCEP1/2, its recruitment to chromosomes occurs independently of ZSL or SCEP1/2, as evidenced by the tremendous axial associations of ZYP1 in *zsl* or *scep1* mutants (Figure 3). Conversely, the chromosome loading of ZSL and SCEP1/2 is strictly dependent on ZYP1 (Figure 6). These results suggest that the association of TF protein ZYP1 to the axis/LEs is likely the initial step of SC polymerization, while the CE proteins is required for ZYP1 zipping through facilitating and/or stabilizing the TF interdigitating connection and extension along co‐aligned axes/LEs to achieve tight synapsis, following a hierarchical assembly mechanism of TF (ZYP1)→ZSL→SCEP1/2 cascade (Figure 9). This is similar to the situation in mammals where the TF protein SYCP1 is first recruited to the axes/LEs and its tight assembly and elongation is reinforced by recruitment of CE proteins (i.e., SYCE3, SYCE1–SIX6OS1, and SYCE2–TEX12) [29, 37].

Super‐resolution microscopy localizes ZSL at the SC midline, which forms linear thread‐like signals and co‐localizes with the N‐terminal domain of ZYP1, placing ZSL as a bona fide CE component. Coincidentally, during the progression of our study, two recent work identified SCEP3 as a CE protein in *A. thaliana*, which is the homolog of ZSL and is similarly also required for SC assembly [19, 21]. Nevertheless, while Feng et al. [19] observed a linear localization pattern for SCEP3 at pachytene, Seear et al. [21] reported a pattern of discontinuous stretches. Moreover, the localization dependency among known TF and CE proteins shows certain similarities between *A. thaliana* and *B. napus*, for example, in both species, chromosome loading of ZYP1 and SCEP3/ZSL is independent of SCEP1/2, but SCEP1/2 recruitment depends on ZYP1 and SCEP3/ZSL [19].

However, a sharp difference emerges. In *A. thaliana*, ZYP1 loading is dependent on SCEP3, whereas SCEP3 recruitment is unaffected by ZYP1 [19]. While no SCEP3 signal was observed before synapsis in wild‐type *A. thaliana* [19], Seear et al. [21] reported that *A. thaliana* SCEP3 already forms numerous foci on the axis during leptotene, which is earlier than ZYP1, and that these SCEP3 foci translocate from the axis to the SC central region at zygotene. In contrast, in *B. napus*, ZYP1 recruitment precedes ZSL and is required for ZSL loading. These observations likely suggests that in *A. thaliana*, the CE protein SCEP3 is likely the initial CR component to be recruited between co‐aligned axes/LEs to initiate SC assembly, which might promote the subsequent recruitment of ZYP1 and SCEP1/2. Conversely, in *B. napus*, ZYP1 appears to be the first CR protein recruited independent of other CE proteins, resembling the mechanism in mammals, where the TF protein SYCP1 is initially loaded onto the axes/LEs and its assembly and elongation are reinforced and stabilized by the subsequent incorporation of CE proteins (e.g., SYCE3, SYCE1–SIX6OS1, and SYCE2–TEX12) [29, 37]. Whether this divergence reflects a species‐specific difference or arises from polyploidy remains to be determined.

### 3.2 ZSL Is a Molecular Adapter Essential for SC Central Region Assembly in *B. napus*

In *A. thaliana*, mutation of either ZYP1 or SCEP1/2 abolishes the CR assembly of the SC, yet no direct connector between them has been identified. It is unclear whether SCEP3 would fulfil this role in *A. thaliana*, particularly given that its chromosome loading is independent of both ZYP1 and SCEP1/2 [17, 19]. Our study provides mechanistic insights into how ZYP1 and SCEP1/2 proteins assemble to form the functional CR of the SC in *B. napus*. We found that ZSL directly interacts with both ZYP1 and SCEP1/SCEP2 heterodimer, and that its presence mediates the formation of ZYP1‐ZSL‐SCEP1/2 complex. Further analysis revealed that ZSL binds to the N‐terminal part of ZYP1 through its C‐terminal α‐helical domain, which is also responsible for interaction with SCEP1/2 heterodimer. The same interaction for the C‐terminal SCEP3 coiled‐coil and N‐terminus of ZYP1 was also identified in *A. thaliana* [19, 21]. These findings support a model where ZSL facilitates CR assembly by linking the N‐terminal parts of TFs (ZYP1) to SCEP1/2 heterodimers, thereby stabilizing the overall CR assembly, providing a plausible structural basis for SC nucleation and elongation (Figure 9). This explains why ZYP1 forms diffuse axial associations but fails to polymerize in *zsl* and *scep1* mutants in *B. napus*. Although the situation remains unclear in *A. thaliana*, the wide presence of ZSL/SCEP3 homologs in angiosperms likely suggests that this scaffold mechanism may be an ancestrally conserved feature of plant meiosis. Notably, Seear et al. [21] recently also established the direct interaction between SCEP3 and SCEP1/2 via Y2H, and their binding interface was mapped to the C‐terminal 480‐801 aa of SCEP3. They further revealed that two separate coiled‐coils (487–579 and 714–801 aa) within SCEP3 C‐terminus show positive interactions with SCEP1/2. This is largely consistent with our observation for ZSL where the C‐terminal coiled‐coil (601–811 aa) are responsible for its binding to SCEP1/2 in *B. napus*.

### 3.3 Functional Significance of SC Integrity in Polyploid Meiotic Stability

In polyploid species where multiple closely related chromosomes coexist, one chromosome has more than one potential pairing partner, which imposes a major challenge to meiotic stability. While the detailed evolutionary mechanisms for meiosis adaptation remain largely unclear, recent work in autotetraploid *A. arenosa* and allotetraploid *B. napus* support that SC plays key roles in stabilizing homologous pairing while suppressing undesirable homoeologous/multivalent interactions [7, 8]. The meiotic defects of *zsl* mutants resemble those of *zyp1* mutants in *B. napus*, including multivalent chromosome pairing, extensive chromosome entanglements, and pronounced chromosome fragmentation, indicating that ZSL‐mediated SC assembly is necessary for maintaining the diploid‐like fidelity of homologous interaction and DSB repair, which are essential for fertility and genome stability in polyploids. However, *B. napus* plants lacking ZYP1 generally exhibit more severe meiotic defects in the occurrence of multivalent associations, chromosome entanglements, and fragmentation [8]. This suggests that, in the absence of ZSL, the chromosome axis‐associated ZYP1 still plays a role in restricting ectopic chromosome interactions. Notably, despite the similar loss of CO assurance and interference, such defects were not observed in diploid *A. thaliana zyp1* and *scep1/2/3* mutants, suggesting a more crucial role of the SC in an allotetraploid context. Together, these observations reinforce the view that during early polyploid establishment, modifications to SC assembly dynamics are central to their adaptation against meiotic instability.

### 3.4 Conserved Impact of SC Disruption on CO Frequency and Interference

Our cytological data and genome‐wide recombination mapping shows that ZSL loss increases CO number by ∼100% in both males and females, erasing the heterochiasmy in *B. napus*. These effects mirror those of SC disruption in diploid *A. thaliana*, where the absence of ZYP1, SCEP1, or SCEP2 increases class I COs (∼50%) and abolishes interference [17, 18, 20, 22]. Similar effects for the loss of SCEP3 in *A. thaliana* was recently reported [19, 21]. Notably, in *A. thaliana scep3*, CO frequencies increase primarily at interstitial and sub‐telomeric chromosome regions, whereas pericentromeric regions remain suppressed or exhibited a reduction. In contrast, *B. napus zsl* mutants show also an increased CO formation at regions closer to centromeres. Despite the elevation in total CO formation, CO assurance is compromised in all CR mutants of *A. thaliana* (*zyp1* and *scep1/2/3*) and *B. napus* (*zyp1* and *zsl*), supporting a conserved role of the SC in obligate CO formation. While ZYP1 is loaded onto chromosomes in the absence of ZSL in *B. napus*, the elimination of interference in *zsl* mutants suggests that a wildtype‐like intact CR is a prerequisite for the long‐range communication of recombination intermediates along paired chromosomes, which enforces CO interference and limits CO number. Intriguingly, compared to males, the disproportionate increase in female COs upon SC disruption in both *A. thaliana* and *B. napus* supports the idea that SC‐dependent interference mechanism is inherently more robust in female meiosis, rendering a relatively larger sensitivity of CO formation to SC perturbation. From an applied perspective, modulating CO number and distribution through SC engineering has significant breeding potential. In polyploid crops such as *B. napus* and wheat, targeted SC component manipulation could be used to unlock genetic variation while maintaining pairing fidelity or conversely to increase the homoeologous pairing and recombination for wild hybridization breeding [8].

### 3.5 Role of SC in HEI10 Dynamics

In recent years, a quantitative HEI10 coarsening model has been proposed to mechanistically explain class I CO patterning, offering a concrete molecular explanation for CO interference [38, 39]. In this framework, HEI10 is treated as a conserved physical quantity that diffuses along synapsed chromosomes, progressively coarsening into a limited number of large foci that designate CO sites. While this model provides an elegant and predictive explanation for CO interference, it may oversimplify its function by abstracting it from its biochemical activities. Importantly, this model does not explicitly incorporate the dependence of HEI10 localization on other meiotic factors or chromosome structure, such as ZMM proteins and the chromosome axis, which very likely provide the molecular basis for HEI10 localization and redistribution thus critically shaping its dynamics and CO interference [32]. In this HEI10 coarsening model, the SC plays a central role by enabling long‐range diffusion and competition of HEI10 along fully synapsed homologous chromosomes, thereby enforcing CO interference. This view is supported by the observations that loss of CR components, including ZYP1 and SCEP1/2, leads to a pronounced increase in HEI10 foci at late pachytene in both *A. thaliana* and *B. napus* [8, 17, 18, 20]. Intriguingly, although CO numbers are elevated in *A. thaliana scep3* mutants, the amount of HEI10 foci at late pachytene remain unchanged compared to that in wildtype [19]. This contrasts with the dramatic increase of HEI10 foci in *B. napus zsl* mutants, indicating possibly the species‐dependent role of ZSL/SCEP3 in regulating HEI10 coarsening. Such differences may point to evolutionary divergence in how CR components modulate HEI10 dynamics, despite their conserved roles in SC assembly.

Generally, although it is clear that the SC is a key regulator of HEI10 focus number and spatial distribution along chromosomes, its effect on the total amount of chromosome‐associated HEI10 protein remains poorly understood. Because focus number does not necessarily correlate with protein abundance, quantitative measurements of HEI10 levels on chromosomes at different prophase I stages, particularly in CR‐defective mutants, will be essential to fully assess how the SC controls HEI10 dynamics and CO outcomes. Another layer of complexity arises from the observation that loss of CR components universally leads to persistent chromosome association of ASY1, a HORMA‐domain protein that acts as a key scaffold and regulator of meiotic recombination. The extent to which defective ASY1 removal contributes to increased CO formation and the loss of interference in SC‐deficient backgrounds remains unresolved [8, 22]. Finally, although SC disruption consistently increases CO numbers and abolishes interference, it is still unclear whether these effects reflect a direct role of the SC in enforcing interference or arise indirectly through affecting the process of homologous recombination. Disentangling these possibilities will be critical for a comprehensive understanding of how the SC governs CO patterning.

In conclusion, we have identified ZSL as a molecular adapter linking TFs to CE proteins in the plant SC, essential for CR assembly, homologous synapsis, and recombination patterning in polyploid meiosis. Despite the establishment of ZSL's molecular role, several questions remain. The detailed organization of the ZYP1–ZSL–SCEP1/2 complex within the CR awaits high‐resolution imaging and structural dissection, and whether ZSL interacts with other CE‐associated proteins or recombination regulators (e.g., ZMM proteins) is unknown. Moreover, the mechanisms by which SC integrity enforces interference and sex‐specific CO patterns merit further mechanistic dissection.

## 4 Experimental Section

### 4.1 Plant Materials and Growth Condition

The spring‐type *B. napus* varieties *Westar* or *J9707* were used as the wild‐type reference in this study. The *heim3*/*zsl1‐1* (*Westar*), *heim3/zsl1‐2* (*Westar*), *heim3/zsl1‐3* (*J9707*), and other *heims* (*Westar*) mutants were generated by CRISPR/Cas9 gene editing technique. The rapeseed plants were grown in greenhouse with a condition of 16 h light at 22°C and 8 h dark at 20°C at Huazhong Agricultural University, Wuhan, China.

### 4.2 Anther Isolation and Collection

For the temporal transcriptome analysis of *B. napus* anthers, we collected three biological replicates at eight development stages. Samples from at least 10 wild‐type plants were collected for each replicate. Briefly, flower buds at early flowering stage were collected and placed on ice. After dissection, four anthers, which have longer stamen filaments and are at a similar developmental stage, were put into cold water on a microscope side. Following the anther length measurement using the optical ruler under a stereoscope, one anther was softened in a 1.5 mL microtube containing 1 mol/L HCl at 60°C for 1 min. Subsequently, the anther was rinsed in water and crushed completely in 20 µL Carbol fuchsin staining solution on a slide. After putting the coverslip, the slide was observed under a light microscope to determine the developmental stage. The remaining three anthers were placed in a microtube and quick‐frozen in liquid nitrogen. Anthers of similar length and at the same developmental stage from at least ten plants grown in the same batch were pooled as one biological replicate. Three independent replicates were collected from plants grown at different times.

### 4.3 RNA Sequencing

Total RNA was extracted using RNAprep Pure Polysaccharide and Polyphenol Plant Total RNA Extraction Kit (Centrifuge Column Type) Catalog Number: DP441 (TianGen, China). Use the Fast RNA‐seq Lib Prep Kit V2 (ABclonal, China, Cat. No. RK20306) kit for library construction. The cDNA library was size‐selected to approximately 370–420 bp using AMPure XP beads (Beckman Coulter, USA), followed by PCR amplification and another round of purification with AMPure XP beads to obtain the final library. After the library was constructed, it was first quantitatively analyzed using a Qubit 2.0 Fluorometer (Invitrogen, Qubit 3.0, USA) for preliminary quantification. Subsequently, the insert size of the library was detected using an Agilent 5400 (Agilent, Agilent 5400, USA). Once the insert size met the expected criteria, the library's effective concentration was accurately quantified using an Ouch q‐PCR system (CFX96 BIO‐RAD, USA) to accurately quantify the effective concentration of the library. Subsequently, Illumina NovaSeq x plus sequencing was performed, generating 150 bp paired‐end reads. All of the above operations are performed by Novogene Bioinformatics Technology Co. Ltd.

### 4.4 Read Mapping and Expression Quantification

Raw RNA sequencing reads were processed with fastp (v0.23.4) using default settings to assess read quality and remove barcode adaptors and low‐quality reads [40]. Then, the filtered reads were compared to the *Brassica napus* reference genome using hisat2 (v2.2.1) with default settings [24, 41]. Reads or fragments from BAM files are counted using the Rsubread (v2.20.0) package “featureCounts” function [42]. TPM was then calculated as a measure of gene expression abundance. The average TPM value for biological duplication was used as the expression level of genes at different stages of development.

### 4.5 Pearson Correlation Coefficient and PCA

Log_2_(TPM+1) was used by psych (v2.5.6) (https://CRAN.R‐project.org/package=psych) to calculate the Pearson correlation coefficient between biological replicates. Log_2_(TPM+1) was used by sklearn (v1.3.2) package PCA class to analysis of PCA between biological replicates [43].

### 4.6 Clustering of Gene Expression Profiles

The 15,000 genes with the largest coefficient of variation (CV) in expression at different stages of development were determined by ClusterGVis (v0.1.3) (https://github.com/junjunlab/ClusterGVis) (the “mfuzz” method) clusters into 10 significantly discrete clustering modules. The Pearson correlation coefficients were calculated by psych (v2.5.6) package (https://CRAN.R‐project.org/package=psych) among genes in the C3 cluster, and gene pairs with Pearson's coefficients > 0.95 or < −0.95 and *p*‐values < 0.05 were identified as highly correlated gene pairs. Use the graph_from_data_frame function of the igraph (v2.0.3) (https://igraph.org) package to build an undirected network for highly correlated gene pairs, and use Cytoscape (v3.10.3) to visualize the correlation network [44].

### 4.7 Plasmid Construction

To generate *heim1* to *heim13* mutants, two sgRNAs were designed for each gene using the CRISPR‐P2.0 (http://crispr.hzau.edu.cn/CRISPR2) (Figure S2). The sgRNAs were synthesized and inserted into the binary vector *PKSE401* that contains the guide RNA scaffold and Cas9 expression cassettes, using the golden gate assembly method [45].

For the yeast two‐hybrid assays, *BnaA07.ZYP1A^FL^‐BD*, *BnaA07.ZYP1A^1‐300aa^‐BD*, *BnaA07.ZYP1A^301‐679aa^‐BD*, *BnaA07.ZYP1A^680‐876aa^‐BD*, *BnaA05.SCEP1^FL^‐BD*, *BnaA05.SCEP1^FL^‐AD*, *BnaC07.SCEP2^FL^‐BD*, *BnaC07.SCEP2^FL^‐AD*, *BnaA03.ZSL^FL^‐AD*, *BnaA03.ZSL^FL^‐AD*, *BnaA03.ZSL^1‐300aa^‐AD*, *BnaA03.ZSL^301‐600aa^‐AD*, and *BnaA03.ZSL^601‐811aa^‐AD* constructs were generated. The CDS sequences of BnaA07.ZYP1A^FL^, BnaA05.SCEP1^FL^, BnaC07.SCEP2^FL^, and BnaA03.ZSL^FL^ were amplified by PCR with primers flanked by attB recombination sites and subcloned *into pDONR223* vector by Gateway BP reactions. The constructs for BnaA07.ZYP1A and BnaA03.ZSL truncated proteins were generated by truncating these full‐length entry vectors through SLICE reaction. All these entry clones were subsequently integrated into the destination vectors *pGADT7‐GW* or *pGBKT7‐GW* vectors by Gateway LR reactions.

For the split GAL4 RUBY assays [31], *BnaA07.ZYP1A^FL^‐VP16*, *BnaA07.ZYP1A^1‐300aa^‐VP16*, *BnaA05.SCEP1^FL^‐VP16*, *BnaC07.SCEP2^FL^‐VP16*, *BnaA03.ZSL^FL^‐VP16*, *BnaA03.ZSL^601‐811aa^‐VP16*, *BnaA07.ZYP1A^FL^‐GAL4*, *BnaA07.ZYP1A^1‐300aa^‐GAL4*, *BnaA05.SCEP1^FL^‐GAL4*, *BnaC07.SCEP2^FL^‐GAL4*, *BnaA03.ZSL^FL^‐GAL4*, *BnaA03.ZSL^601‐811aa^‐GAL4* constructs were generated. The corresponding CDS sequences were amplified by PCR using primer pairs containing different endonuclease cutting sites. Following enzyme digestion, the CDS sequences were ligated into VP16 or GAL4 vectors. For the expression construct of *BnaA03.ZSL^FL^_*pGWB602, BnaA03*.ZSL^601‐811aa^_pGWB602*, and *BnaC07.SCEP2^FL^_pGWB602*, the corresponding CDS sequences were first inserted into *pDONR221* vector by Gateway BP reactions, and then integrated into the destination vector *pGWB602* by Gateway LR reactions. The primers for creating these vectors are listed in Table S5.

### 4.8 Plant Transformation and Genotyping

The agrobacterium‐mediated transformation of *B. napus* was carried out as previously described using hypocotyl as explants [46]. To identify the mutations induced by CRISPR/Cas9, each copy of *ZSL* and other *HEIM* genes was amplified by PCR using the gene‐specific primers. The PCR fragments were sequenced by the gene‐specific primers. The primers used are listed in Table S5.

### 4.9 Pollen and Tetrad Staining

The pollen staining was performed by dipping open flowers into Peterson staining solution on microscopic slides, which were then heated at 80°C for 10 min to increase the contrast between dead and viable pollen grains [47]. For tetrad analysis, the flower buds of appropriate size (1.3–1.7 mm) were dissected, and isolated anthers were softened in 1 mol/L HCl at 60°C for 1 min. Subsequently, anthers were rinsed in distilled water and squashed in orcein staining solution on slides for microscopic examination. Images were captured using a SOPTOP optical microscope ex30 equipped with a color camera.

### 4.10 Antibody Generation

The polyclonal antibodies against *B. napus* ZSL, ZYP1‐N, ZYP1‐C, SCEP1, and SCEP2 were produced by MabStar, Wuhan, China (http://mabstar.com.cn). Briefly, the coding regions of BnaC06.ZSL (1‐225 aa), BnaA07.ZYP1B (1‐390 aa), BnaA07.ZYP1B (503‐876 aa), BnaC05.SCEP1 (1‐180 aa), and BnaA06.SCEP2 (92‐223 aa) were amplified and inserted into the *pET‐32a* prokaryotic expression vector. Recombinant proteins were produced and purified from BL21(DE3) *Escherichia coli* bacteria, which were used as antigens to immunize rabbits, rats, or chicken. After three‐time immunization, antibodies were purified from the antisera using antigen‐based affinity purification. The other antibodies used in this research were produced previously [32].

### 4.11 Cytological Analysis

Chromosome spreading analysis was carried out as described previously (cite [32] Plant Cell). Flowers at early flowering stage were collected and fixed in ethanol: acetic acid (3:1, v/v) fixative for 72 h at 4°C, and then stored at −20°C. To prepare the chromosome spreads, the entire flower was washed in sodium citrate buffer (pH 4.5) for three times (10 min each) to remove the fixative. Flower buds of appropriate size (∼1–2 mm) were selected. From each bud, one anther was taken and squashed in Carbol‐fuchsin solution to determine the meiotic stage under a light microscope. Next, the remaining five anthers were placed in an enzyme solution (3% cellulose, 3% macerozyme, and 5% snailase in 50 mm citrate buffer pH 4.5) to digest for 50 min. Subsequently, single digested anthers were transferred onto a microscopic slide, 15 µL of 60% ice‐cold acetic acid was added, and anthers were then macerated into a homogenous mixture using a bent needle. An additional 15 µL of 60% ice‐cold acetic acid were added and the liquid mixture was spread immediately by circular stirring on a heating plate at 45°C until the liquid was largely evaporated. The slide was rinsed once with ice‐cold fixative (3:1 v/v ethanol: acetic acid), drained shortly on the heating plate, and dried at room temperature for 1 h. The slides were then stained with DAPI for observing meiotic chromosome behaviors. Images were captured using a SOPTOP fluorescence microscope RX50 equipped with a monochrome camera.

For immunostaining, the slides with spread chromosomes were immerged into a glass jar with 10 mm citrate buffer pH 6.0, and heated using a microwave until slight boiling. Immediately transfer the slides to 1× phosphate‐buffered saline with Triton X‐100 detergent (PBST) solution (0.1% (v/v) Triton X‐100) at room temperature for 5 min. Next, the samples were blocked by covering the slides with goat serum for 1–2 h, then the slides were incubated with the corresponding combination of primary antibodies at 4°C for 24–36 h. After washing the slides for three times in PBST, the slides were incubated with fluorescein conjugated secondary antibody (ThermoFisher) at 4°C for 24 h. After washing the slides for three times in PBST, the antifade DAPI solution (VECTASHIELD) was added onto the slides to stain DNA. Images were captured using a SOPTOP fluorescence microscope RX50 equipped with a monochrome camera.

### 4.12 Yeast Two‐Hybrid Assay

For the yeast two‐hybrid assays, the relevant vector combinations were co‐transformed into auxotrophic yeast strain AH109 using the polyethylene glycol/lithium acetate method according to the manufacturer's manual (Clontech). Positive clones were selected on the plate of double (–Leu–Trp) synthetic dropout medium. Subsequently, yeast cells from positive clones were dotted on the plates of double (–Leu–Trp) and triple (–Leu–Trp–His) synthetic dropout media. Images were acquired after incubating the plates at 28°C for 2 or 3 days.

### 4.13 Split GAL4 RUBY Assay

For the split GAL4 RUBY assays, the relevant constructs were transformed into the agrobacterium strain GV3101. The agrobacteria were incubated at 28°C and harvested at OD_600_ ≈ 0.8. The pellets were resuspended in the infiltration buffer (10 mm MES, 10 mm MgCl_2_, and 150 mm acetosyringone). The relevant combinations of agrobacteria were infiltrated into tobacco (*N. benthamiana*) leaves, which were then kept in dark for 12 h, and put back to normal growth condition for 3 days. The leaves were decolorized until white in absolute ethanol solution and images were taken.

### 4.14 Genome‐wide Mapping of Male and Female COs

To generate male and female CO mapping populations, *zsl‐1* (*Westar*) was crossed with *zsl‐3* (*J9707*), and F1 hybrids of the WT and *zsl* were crossed as a male or female with the WT (*Westar*). Total DNA samples were extracted from the leaves of the four resulting backcross populations (WT male, 49 plants; WT female, 50 plants; *zsl‐1 zsl‐3* male, 48 plants; *zsl‐1 zsl‐3* female, 48 plants), and then sent for deep sequencing. Paired‐end 150 bp whole‐genome resequencing was performed on the Illumina HiSeq Nova platform, with library construction and sequencing services provided by Genoseq and BioMarker Technologies.

Raw sequencing reads were quality‐controlled and filtered using fastp v0.25.0 [40] to obtain high‐quality clean data. The clean data from each sample were aligned to the Westar v0 reference genome [24] using BWA v0.7.19‐r1273 [48] with default parameters. The resulting BAM files were sorted with SAMtools v1.22 [49], and PCR duplicates were removed using Picard. Genome‐wide variant calling (SNPs and InDels) was performed with GATK v4.0.3.0 [50]. Markers that were polymorphic and homozygous between the Westar and J9707 parents were selected for CO identification. Meiotic COs were detected using a sliding window approach with a 50 kb window size and 25 kb step size. A double CO event was defined as being supported by at least 500 kb of continuous genomic interval. The information of all COs identified is provided in Tables S6–S9.

For CO distribution analysis, a systematic scan was performed along each chromosome using 1 Mb windows with a 50 kb sliding step to characterize the distribution pattern of COs. The midpoint of the 1 Mb window was plotted on the graph to indicate the recombination rate in this window. Centromeric regions were identified based on the Westar v0 reference genome using CentIER v2.0 [51] and depicted on CO distribution maps. The value of CoC was calculated to quantify CO interference. Briefly, each chromosome was divided into fixed‐size intervals. CO events were assigned to intervals based on their midpoint positions, and the CO frequency per interval was computed. The observed number of double COs for pairs of intervals on the same chromosome was counted. The expected number was calculated under the assumption of independent CO distribution. The CoC was then defined as the ratio of observed to expected values.

### 4.15 Accession Numbers

The sequence data of key genes are available in the *Brassica napus* multi‐omics information resource database BnIR, with the following gene accession numbers: *BnaA01.ZSL* (BnaA01G0015100WE), *BnaA03.ZSL* (BnaA03G0455300WE), *BnaC01.ZSL* (BnaC01G0088700WE), *BnaC06.ZSL* (BnaC06G0182500WE), *BnaA07.ZYP1A* (BnaA07G022800WE), *BnaC06.ZYP1A* (BnaC06G0283400WE), *BnaA07.ZYP1B* (BnaA07G0369500WE), *BnaC06.ZYP1B* (BnaC06G0455800WE), *BnaA05.SCEP1* (BnaA05G0272300WE), *BnaC05.SCEP1* (BnaC05G0317600WE), *BnaA06.SCEP2* (BnaA06G0377800ZS), *BnaA06.SCEP2* (BnaA06G0377900ZS), and *BnaC07.SCEP2* (BnaC07G0312900ZS).

### 4.16 Statistical Analysis

The Mann–Whitney *U* test and one‐way ANOVA followed by Tukey's multiple comparison test were performed using GraphPad Prism 8.0.2 software. The calculation of the mean and standard deviation was done using Microsoft Excel. The details of statistical method, data presentation, sample size (*n*), and probability value (*P*) can be found in relevant figure legends.

## Author Contributions

C.Y. conceived this research. M.G., F.C., C.L., H.Z., J.Z., Y.Z., C.L., Y.Z., Z.G., X.Y., J.Y., and L.C. performed the experiments. H.Z. provided supervision and guidance. M.G., S.J., G.X., and C.Y analyzed the data. C.Y. and M.G. wrote the manuscript.

## Conflicts of Interest

The authors declare no conflicts of interest.

## Supporting information




**Supporting File**: advs74682‐sup‐0001‐SuppMat.docx.

## Data Availability

The data that support the findings of this study are available in the supplementary material of this article.
