# Supplementary material for: ZSL Orchestrates Synaptonemal Complex Assembly as a Central Region Scaffold to Ensure Synapsis Fidelity and Crossover Control in Polyploid Meiosis
Source: Adv Sci (Weinh). 2026 Mar 4;13(27):e21496. doi: 10.1002/advs.202521496 (PMC13170185; doi:10.1002/advs.202521496)
Supplement: Supplementary file 1 — Supporting File: advs74682‐sup‐0001‐SuppMat.docx. [file ADVS-13-e21496-s001.docx]

Supporting Information

ZSL ORCHESTRATES SYNAPTONEMAL COMPLEX ASSEMBLY AS A CENTRAL REGION SCAFFOLD TO ENSURE SYNAPSIS FIDELITY AND CROSSOVER CONTROL IN POLYPLOID MEIOSIS

*Miaowei Geng, Shaochen Jia, Fei Cao, Cuiping Liu, Heshuang Zhang, Gang Xu, Jixin Zhuang, Yashi Zhang, Can Liu, Yuhang Zhao, Zilin Guo, Xinjie Yuan, Jiaqing Yang, Lei Chu, Bowei Cai, Hu Zhao, and Chao Yang**

**Supplemental figures and figure legends**

**Supplemental figure 1. Sample correlation and principal component analysis of the anther transcriptome in eight development stages of *Brassica napus*.**

A: Heatmap of Pearson correlation coefficients among different biological replicates.

B: Principal component analysis of different biological replicates.

**Supplemental figure 2. Generation and identification of *heim1*, *heim3* (*zsl*), *heim5*, *heim6*, *heim9*, *heim11*, *heim12*, and *heim13* mutants using CRISPR-Cas9.** Two sgRNA targets were designed for each gene. The blue letters/dashed lines indicate the insertion or deletion mutations. Two independent mutant lines were generated for each gene except for *HEIM9* having only one mutant line.

**Supplemental figure 3. Vegetative growth of *heim1*, *heim3* (*zsl*), *heim5*, *heim6*, *heim9*, *heim11*, *heim12*, and *heim13* mutants.** Bars: 10 cm.

**Supplemental figure 4. ZSL phylogenetic tree in plants.** The evolutionary analysis was based on *B. napus* ZSL homologous sequences from PSI blast and constructed using the Maximum Likelihood method and JTT matrix-based model. The bootstrap consensus tree inferred from 1000 replicates is taken to represent the evolutionary history of the taxa analyzed. The red line highlights a species belong to mosses.

**Supplemental figure 5. Evaluation of meiotic duration and specificity validation of ZSL and SCEP1 antibodies.**

A: Correlation between meiotic stages and anther sizes in WT (*Westar*) and *zsl* mutants.

B: Protein alignment of four ZSL copies. Red rectangle highlights the N-terminal part of BnaC06.ZSL (1-225 aa) used for antibody production.

C: Co-immunostaining of ZSL with ASY1 in male meiocytes of *B. napus* *zsl-1* and *zsl-2* mutants at pachytene-like stage.

D: Co-immunostaining of SCEP1 with ASY1 in male meiocytes of *B. napus* *scep1-1* mutants at pachytene-like stage. Bars: 5 μm.

**Supplemental figure 6. DSB formation is not affected in *zsl* mutants.**

A: Immunolocalization of DMC1 in male meiocytes of *B. napus* WT (*Westar*), *zsl-1*, and *zsl-2* mutant plants at early prophase I. Bars: 5 μm.

B: Quantification of relative DMC1 signal intensity shown in (A). Error bars indicate mean ± SD. Tukey’s multiple comparison test (ns, no significance).

**Supplemental figure 7. Generation and phenotypic analysis of *scep1* mutants in *B. napus*.**

A: Generation and identification of *secp1-1* and *scep1-2* mutants using CRISPR-Cas9. Two sgRNA targets were designed. No mutation was identified from target 1 site. The red letters/dashed lines indicate the insertion or deletion mutations from target 2 site. Two independent mutant lines were generated.

B: Main silique branches of WT, *scep1-1*, and *scep1-2* mutant plants. Bars: 5 cm.

C: Quantification of the number of seeds per silique in WT, *scep1-1*, and *scep1-2* mutant plants. At least 15 siliques were dissected and counted for each genotype. Error bars indicate mean ± SD. Tukey’s multiple comparison test, *** *P* < 0.001.

D: Pollen viability in WT, *scep1-1*, and *scep1-2* mutant plants. At least 4000 pollen grains were counted from different flowers for each genotype. Error bars indicate mean ± SD. Tukey’s multiple comparison test, *** *P* < 0.001.

E: Staining of male meiotic products at tetrad stage in WT, *scep1-1*, and *scep1-2* mutant plants. Bars: 5 μm.

F: Pie charts depicting the proportion of balanced tetrad, unbalanced tetrad, polyad, triad, dyad, and monad in WT, *scep1-1*, and *scep1-2* mutant plants.

Error bars in (C) and (D) indicate mean ± SD. Tukey’s multiple comparisons test, *** *P* < 0.001.

**Supplemental figure 8. Experimental controls for the protein-protein interaction assays.**

A: Yeast two-hybrid assay showed no autoactivation for ZYP1, SCEP1, SCEP2, ZSL, and the truncated versions.

B: Split GAL4 RUBY assay showed no autoactivation for ZYP1, SCEP1, SCEP2, ZSL, and the truncated versions.

**Supplemental figure 9. Generation and phenotypic analysis of *zsl-3* mutants in *B. napus* variety *J9707*.**

A: Generation and identification of *zsl-3* mutants using CRISPR-Cas9 in *B. napus* variety *J9707*. Two sgRNA sequences (blue and black arrowheads) were designed, which targets the homologous copies on chromosome A03 and C06 or A01 and C01, respectively. The blue letters/dashed lines indicate the insertion or deletion mutations.

B: Pollen staining of WT (*J9707*) and *zsl-3* (*J9707*) mutant plants. Four plants were examined for each genotype. Bars: 100 μm.

C: Pollen viability in WT (*J9707*) and *zsl-3* (*J9707*) mutant plants. At least 4000 pollen grains were counted from different flowers for each genotype. Error bars indicate mean ± SD. Student’s t test, *** *P* < 0.001.

D: Staining of male meiotic products at tetrad stage in WT (*J9707*) and *zsl-3* (*J9707*) mutant plants. Bars: 5 μm.

E: Pie charts depicting the proportion of balanced tetrad, triad, and dyad, and monad in WT (*J9707*) and *zsl-3* (*J9707*) mutant plants.

F: Siliques of WT (*J9707*) and *zsl-3* (*J9707*) mutant plants.

G: Quantification of the number of seeds per silique in WT (*J9707*) and *zsl-3* (*J9707*) mutant plants. At least 20 siliques were dissected and counted for each genotype. Error bars indicate mean ± SD. Student’s t test, *** *P* < 0.001.

**Supplemental figure 10. Distribution of polymorphic markers between the *B. napus* variety *Westar* and *J9707*. The scale for the density distribution of polymorphic markers represents the number of polymorphic markers within 10 kb windows.**

**Supplemental figure 11. Genome-wide CO analysis for all chromosomes with available markers reveals the deficiency in our approach.**

A: The number of COs per gametes for all chromosomes in male and female WT. Each dot indicates an individual BC1 plant. The mean value of each population is indicated at the top and n values represent the number of plants used for analysis.

B: CoC analysis for all chromosomes in male and female WT with a 5-Mb distance between intervals.

C: The number of COs per gamete for each chromosome in male and female WT. Note that since a given CO involves only two of the four chromatids of a recombined bivalent and that a gamete inherits only a single chromatid, the number of COs observed per gamete for each chromosome is on average one-half of the total amount of COs produced during each round of meiosis. In this analysis, only Chr. A04, A07, A08, and A10 highlighted in red show a normal CO frequency consistent with cytological observations.

**Supplemental figure 12. Distribution of COs along chromosome A4, A7 and A8 in male and female WT and *zsl1-1*.** The centromeric regions are indicated by grey shading, respectively. The analysis was done with 1 Mb windows and 50 kb sliding steps.
